# CXCL9 chemokine promotes the progression of human pancreatic adenocarcinoma through STAT3-dependent cytotoxic T lymphocyte suppression

**DOI:** 10.18632/aging.102638

**Published:** 2020-01-08

**Authors:** Hui-Feng Gao, Chien-Shan Cheng, Jian Tang, Ye Li, Hao Chen, Zhi-Qiang Meng, Zhen Chen, Lian-Yu Chen

**Affiliations:** 1 Department of Oncology, Shanghai Medical College, Fudan University, Shanghai 200032, China; 2 Department of Integrative Oncology, Fudan University Shanghai Cancer Center, Shanghai 200032, China

**Keywords:** pancreatic adenocarcinoma, chemokine, CXCL9, CD8+ cytotoxic T cells, STAT3 activation

## Abstract

Chemokines play essential roles in the progression of various human cancers; however, the expression and role of CXC chemokines in pancreatic adenocarcinoma (PAAD) have not yet been identified. The aim of this study is to identify the expression patterns, clinical significance and mechanisms of CXC chemokines in regulating tumour microenvironment of PAAD. Three CXC chemokines, including CXCL5, CXCL9, and CXCL10, were significantly overexpressed in PAAD tissues, which were correlated with the poor survival of the patients. CXCL9/10 was associated with change of immune cell pattern in the tumour microenvironment, and supplementation of CXCL9 in the orthotopic murine PAAD model promoted tumour progression. In particular, CXCL9 reduced the CD8+ cytotoxic T lymphocytes in the tumour microenvironment of PAAD, which could be attributed to the reduced CD8+ T cell proliferation, activation, and secretion of anti-tumour cytokines. *In vitro* treatment of CXCL9 directly led to the suppression of the proliferation, activation, and secretion of anti-tumour cytokines of isolated CD8+ T cells. Inhibition of STAT3 recovered the CXCL9-inhibited proliferation, activation, and secretion of anti-tumour cytokines of CD8+ T cells. Our study indicates CXCL9 as a potential target of immunotherapy in PAAD treatment by regulating the CD8+ T lymphocytes in the tumour microenvironment.

## INTRODUCTION

Pancreatic adenocarcinoma (PAAD), accounting for the most common form of pancreatic cancer, is one of the most malignant solid tumour among various types of tumours. Early diagnosis of PAAD is difficult as no significant sign of diseases could be easily observed, and treatment at the progressive stage of PAAD is not desirable due to the primary resistance of PAAD cells to most of the drugs [1]. The prognosis of PAAD patients is abysmal, with a median overall survival of six months and a 5-year survival rate of less than 5% [1]. The treatment option is minimal. Only 10–15% PAAD patients at very early stage could receive a curative resection, while a large number of patients may receive chemotherapy that gains minimal benefit to the outcome [2, 3], suggesting development of novel treatment targets and strategies is of great importance. Accumulating recent evidence has indicated that the tumour microenvironment of PAAD plays a critical role in the progression and metastasis of the tumour cells. The dynamic composition of the tumour microenvironment, including immune cells, cytokines, fibroblast, blood vessel and extracellular matrix, interact with tumour cells as well as with each other during the progression of preneoplastic pancreatic intraepithelial neoplasia to invasive PAAD [4]. The immunological composition of the tumour microenvironment is the most crucial mediator in PAAD progression. Adaptive T immune cells play differential roles in PAAD. The number of cytotoxic CD8+ T cells [5, 6], which execute surveillance and elimination of tumour cells, predicted a better prognosis of PAAD patients, while the CD4+ T-lymphocytes were prone to exhibit a tumour-promoting effect on PAAD by the secretion of a series of oncogenic cytokines that can primarily affect the capacity of cytotoxic T lymphocytes (CTLs) [7–11]. The innate immune cells, such as tumour-associated macrophages (TAMs) and myeloid-derived suppressive cells (MDSC) delivered suppressive effect to the CTLs immune responses and led to PAAD progression [12, 13]. Identification of targets on immune microenvironment may derive novel strategies in the treatment of PAAD.

Chemokines are a family of small cytokines that can induce directed chemotaxis. Secreted by various types of cells, chemokines could be categorized according to their behaviours and structure characteristics. The CXC chemokines are a group of 17 α-chemokines that delivered multiple physiological and pathological functions. Accumulating studies have suggested that CXC chemokines play essential roles in the initiation and progression of PAAD [14]. Most of the CXC chemokine members could be found in PAAD to promote tumour cell proliferation, angiogenesis, metastasis as well as chemoresistance. Several CXC chemokines were found as regulators of the tumour microenvironment. For example, high expression of CXCL1 could promote recruitment of TAMs into PAAD tissue [15], while CXCL2 led to infiltration of MDSCs into tumour microenvironment [16]. CXCL5 could attract Ly6G+ granulocytes to establish an early metastatic niche of colon cancer in lung tissue [17]. CXCL9, CXCL10, and CXCL11 were found to recruit CD4+ and CD8+ lymphocytes [18–20], while CXCL13 could specifically attract T regulatory and Th2 lymphocytes into the tumour microenvironment [21]. These findings suggest the critical roles of CXC chemokines in the tumour microenvironment; however, systemic investigation on their roles in PAAD remains unavailable.

In this study, we systematically examined the expression and clinical significance of CXC chemokines in PAAD. Expression of CXC chemokines in PAAD tissues and adjacent healthy tissue was retrieved from GEO database. The survival of PAAD patients was collected from GEPIA database. The immune cell patterns of PAAD tissues were retrieved from TIMER database. The correlation was analysed. Murine orthotopic PAAD model was established to examine the effect of selective CXC chemokine on the tumour progression and immune patterns. *In vitro* T cells activation was performed to determine the molecular mechanisms underlying the immunosuppressive action of selective CXC chemokine. Our study suggests a crucial role of CXC chemokines in the tumour microenvironment of PAAD.

## RESULTS

### CXCL9/10 chemokine correlates the prognosis as well as the regulation of tumour microenvironment in PAAD

**Figure 1:**
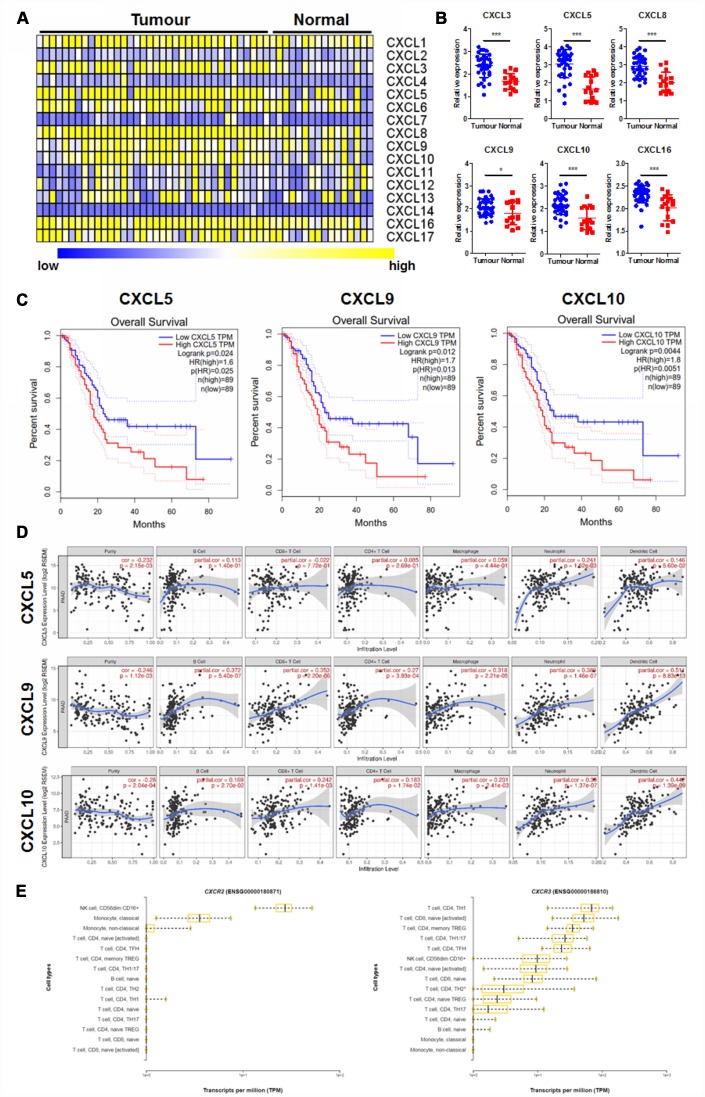
**CXC chemokines expression was correlated with prognosis and immune cell patterns of PAAD.** (**A**) showed the Heatmap expression patterns of CXC chemokines in PAAD extracted from the GEO database (GDS4102); (**B**) showed CXC chemokines with significant changes in expression in PAAD; (**C**) showed CXC chemokines whose expression was correlated with overall survival of PAAD patients; (**D**) showed the correlation of selected CXC chemokines with the immune cell patterns in the tumour microenvironment; (**E**) showed the expression pattern of CXC chemokine receptors, CXCR2, and CXCR5, in different types of immune cells. *p<0.05, ***p<0.001 when compared.

CXC chemokines have been extensively studied for the role in different kinds of cancers and have been suggested to closely related to the tumour angiogenesis and metastasis [23]. However, the roles of CXC chemokines in PAAD was poorly understood. To systemically evaluate the role of different CXC chemokines in PAAD, we first of all retrieved their expression in PAAD tissues and adjacent healthy tissues from GEO dataset (GDS4102, https://www.ncbi.nlm.nih.gov/sites/GDSbrowser?acc=GDS4102). Heatmap of the expression of 16 major CXC chemokines in tumour and normal tissues was established (Figure 1A). Six CXC chemokines out of the 16 were found overexpressed in PAAD tissue compared with its adjacent normal tissue (Figure 1B). To further understand the clinical significance of their differential expression, survival data of CXC chemokines with significant changes was retrieved from GEPIA database (http://gepia.cancer-pku.cn/index.html). Only the expression of three CXC chemokines, including CXCL5, CXCL9, and CXCL10, was significantly correlated with the overall survival of PAAD patients. Overexpression of CXCL5, CXCL9, and CXCL10 predicted with the poor prognosis of the patients (Figure 1C).

Chemokines were believed to play an essential role in regulating the immunological microenvironment of the cancers [24]. To further justify how overexpressed chemokines regulated tumour microenvironment of PAAD, we retrieved the immune cell pattern of PAAD. The correlation of chemokines with immune cell patterns in PAAD was measured. It was shown that CXCL5 has less correlation with immune cell pattern change in PAAD, while CXCL9/10 was significantly correlated with the pattern expression of various immune cells (Figure 1D). As CXCR2 and CXCR3 are the receptors of CXCL5 and CXCL9/10 on immune cells respectively, we further measured their expression in various immune cells of PAAD tissue. While patterns of CXCR2 in immune cells were not consistent with the patterns of immune cells induced by CXCL5, CXCR3 was highly expressed in T cells in the tumour microenvironment of PAAD tissue, which was in agreement with the induced patterns change of immune cells induced by CXCL9/10 (Figure 1E). These results suggested the possible role of CXCL9/10 in the regulation of tumour progression and immune microenvironment in PAAD.

### CXCL9 promotes PAAD tumour progression with altering CD8+ T cells pattern

**Figure 2:**
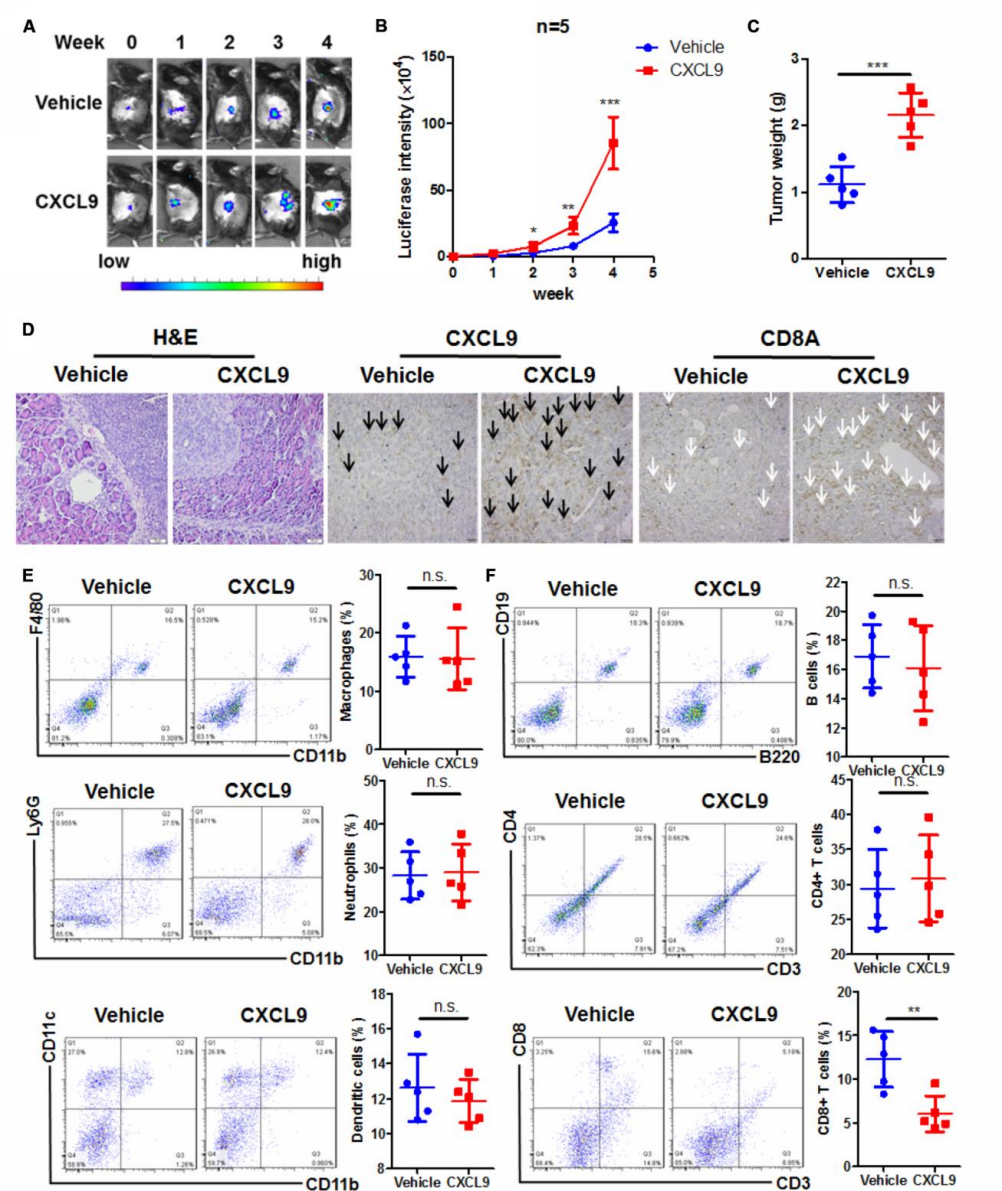
**CXCL9 promoted tumour progression in murine orthotopic PAAD model.** (**A**) showed CXCL9 accelerated tumour growth of orthotopic murine PAAD in mice; (**B**) showed the increased luciferin signals detected in mice was speeded up by CXCL9 treatment; (**C**) showed CXCL9 treatment increased tumour weight of orthotopic murine PAAD; (**D**) showed that CXCL9 treatment led to more aggressive pattern of tumour cells in orthotopic murine PAAD; immunohistological analysis confirmed that CXCL9 treatment could increase the intratumoral CXCL9 level (black arrow)while reduce the CD8+ cytotoxic T cells (white arrow); (**E**) showed that CXCL9 treatment had minimal effect on the pattern of innate immune cells in the tumour microenvironment; (**F**) showed that CXCL9 treatment significantly reduced the CD8+ cytotoxic T cells without affecting other adaptive immune cells in the tumour microenvironment of PAAD. *p<0.05, **p<0.01, ***p<0.001 when compared with control.

The role of CXCL10 in pancreatic cancers seems clear, while that of CXCL9 remains undefined. High expression of CXCL10 in PAAD tumour indicated the poor survival of patients. Mechanistically, CXCL10 delivered immunosuppressive effect and was able to recruit Treg cells, which expressed CXCR3 [25]. CXCL10 was major secreted by pancreatic stellate cells and could be delivered to PAAD tissue to foster an immunosuppressive tumour microenvironment [26]. As CXCL9 and CXCL10 shares same receptor CXCR3 in T lymphocytes, we would like to understand whether the CXCL9 has similar or opposite function with CXCL10 in regulating tumour microenvironment. Murine PAAD cells Panc-2 expressing luciferase reporter were injected to the pancreas of C57BL/6J mice to establish an orthotopic PAAD murine model (Figure 2A). Recombinant murine CXCL9 (10 mg/kg) was intraperitoneally injected three times a week. By measuring the increase of luciferin signals, it was found that treatment of CXCL9 could significant accelerate the growth of PAAD tumour (Figure 2B). At the end of study, pancreas with tumour was dissected out and weighed. The weight of PAAD tumour was potently increased in mice treated with CXCL9 (Figure 2C). Histological analysis showed that the tumour tissue in CXCL9-treated mice seemed more aggressive and active. While clear borderline between the tumour and normal tissues could be observed in mice of control group, dim boundary in the tissue of CXCL9-treated mice was found, suggesting more tumour invasion and infiltration after CXCL9 treatment (Figure 2D). Immunohistochemical analysis confirmed that CXCL9 expression was increased (black arrow) after recombinant protein treatment, while CXCL9 treatment could significantly reduce the peritumoral and intratumoral CD8+ cytotoxic T cells (white arrow) (Figure 2D). Further examination on the immune cell profile of PAAD tissue from control and CXCL9-treated mice found that CXCL9 had minimal effect on the pattern changes of the innate immune cell population, including CD11b+F480+ macrophages, CD11b+Ly6G+ neutrophils and CD11b+CD11c+ dendritic cells (Figure 2E). Treatment of CXCL9 did not change the overall population of B220+CD19+ B lymphocytes or CD3+CD4+ T lymphocytes, while could significantly reduce CD3+CD8+ cytotoxic effector T cells (Figure 2F), which was consistent with the dataset analysis as shown in Figure 1. These findings suggested that CXCL9 promotes PAAD progression and suppresses CD8+ cytotoxic T cells in tumour tissues.

### Suppression of cytotoxic T cells was responsible for the tumour-promoting action of CXCL9

**Figure 3:**
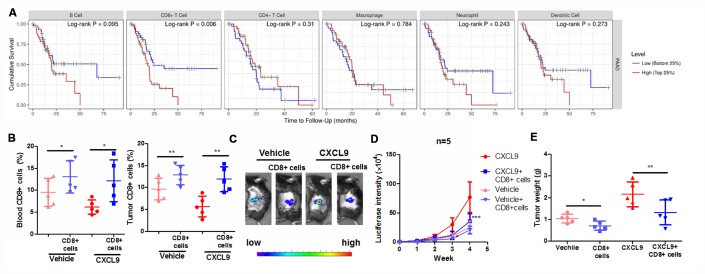
**Suppression of CD8+ cytotoxic T cells was responsible for the tumour-promoting effect of CXCL9.** (**A**) showed the correlation between survival of PAAD with the pattern expression of different immune cells; (**B**) showed that the adoptive transfer of CD8+ cytotoxic T cells successfully increased the peripheral and intratumoral CD8+ cytotoxic T cells in orthotopic murine PAAD model; (**C**) showed that adoptive T cells transfer suppressed the tumour progression induced by CXCL9; (**D**) showed that the increased luciferin signals by CXCL9 were inhibited by adoptive transfer of CD8+ T cells; (**E**) showed that adoptive transfer of CD8+ cytotoxic T cells reduces tumour weight of PAAD in CXCL9-treated mice. *p<0.05, **p<0.01, ***p<0.001 when compared with CXCL9 group.

To further understand the role of the CD8+ cytotoxic T cells in mediating CXCL9-induced PAAD progression, we, first of all, analysed the correlation between the survival of PAAD patients and different immune cell population. By retrieving data from TIMER database (http://cistrome.org/TIMER/). Interestingly, we found that survival of PAAD patients was uniquely correlated with the presence of CD8+ cytotoxic T cells (Figure 3A). As we observed a significant reduction of CD8+ cytotoxic T cells after CXCL9 treatment, we performed adoptive transfer of CD8+ cytotoxic T cells in CXCL9-treated mice to observe if the tumour-promoting action of CXCL9 was associated with the population. CD8+ cytotoxic T cells were isolated from splenocytes and intravenously injected into CXCL9-treated mice. Intravenously injection of CD8+ cytotoxic T cells could successfully replenish the circulating and tumoral CD8+ cytotoxic T cells, suggesting the successful adoptive transfer (Figure 3B). Transfer of CD8+ cytotoxic T cells significantly reduced tumour growth, as indicated by repressed luciferase signal (Figure 3C and 3D). Further analysis of the tumour weight suggested that adoptive transfer of cytotoxic T cells could potently reduce tumour size and tumour weight (Figure 3E). As CXCL9 treatment showed significant suppression on CD8+ population in PAAD tumour, the findings of adoptive transfer suggested that suppression of cytotoxic T cells could be responsible for the tumour-promoting action of CXCL9 in PAAD.

### CXCL9 treatment suppressed *in vivo* activation of cytotoxic T cells

**Figure 4:**
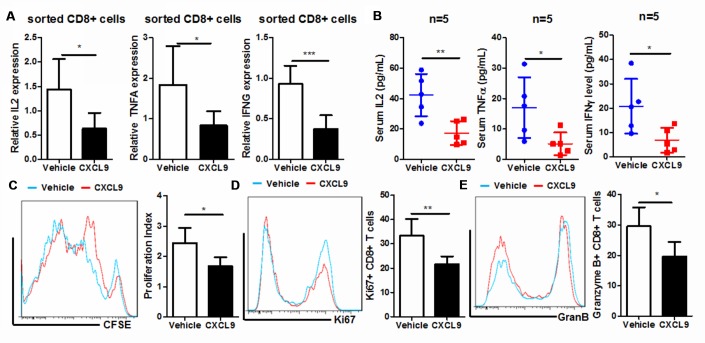
**CXCL9 suppressed *in vivo* proliferation and activation of CD8+ cytotoxic T cells.** (**A**) showed *in vivo* CXCL9 treatment suppressed mRNA expression of anti-tumour cytokines IL2, TNFα, and IFNγ in CD8+ cytotoxic T cells; (**B**) showed that *in vivo* CXCL9 treatment suppressed serum level of anti-tumour cytokines IL2, TNFα, and IFNγ in orthotopic murine PAAD mice; (**C**) showed that *in vivo*, CXCL9 treatment significantly retarded the proliferation of CD8+ cytotoxic T cells; (**D**) showed that *in vivo* CXCL9 treatment significantly repressed expression of proliferation marker Ki67; (**E**) showed that *in vivo* CXCL9 treatment significantly repressed expression of activation marker Granzyme B. *p<0.05, **p<0.01, ***p<0.001 when compared with control.

As the major effector cells that process anti-tumour surveillance in cancer patients, CD8+ cytotoxic T cells were mainly compromised and exhausted by the oncogenic factors in the tumour microenvironment, with a sign of less proliferation, blunt activation and reduced expression of anti-tumour cytokines [27]. To observe if injection of CXCL9 could induce cytotoxic T cell exhaustion, we, first of all, sorted the CD8+ cytotoxic T cells from PAAD tumour tissue of mice. Expression of anti-tumour cytokines, including TNFα, IL2, and IFNγ in sorted CD8+ cytotoxic cells, were measured by quantitative real-time PCR. Treatment of CXCL9 could significantly suppress the expression of anti-tumour cytokine, suggesting the dysfunction of cytotoxic T cells after CXCL9 cells (Figure 4A). The reduced expression of anti-tumour cytokines in CXCL9-treated mice was further proven by the observation that serum level of TNFα, IL2, and IFNγ in CXCL9-treated mice was remarkably suppressed (Figure 4B). CD8+ cytotoxic cells from control and CXCL9-treated mice were then activated with anti-CD3 and anti-CD28 antibodies, and CSFE staining was used to measure the proliferation rate of the cells. CD8+ cytotoxic cells from CXCL9-treated mice significantly had lower proliferation rate (Figure 4C). Intracellular staining of Ki67 and Granzyme B suggested a reduced expression of both proteins in CD8+ T cells from CXCL9-treated mice compared with those from control mice, suggesting the reduced proliferation and activation of the cells. These data suggested that CXCL9 significantly induced *in vivo* exhaustion and dysfunction of CD8+ cytotoxic T cells.

### *In vitro* CXCL9 treatment suppressed CD8+ cytotoxic T cell activation

**Figure 5:**
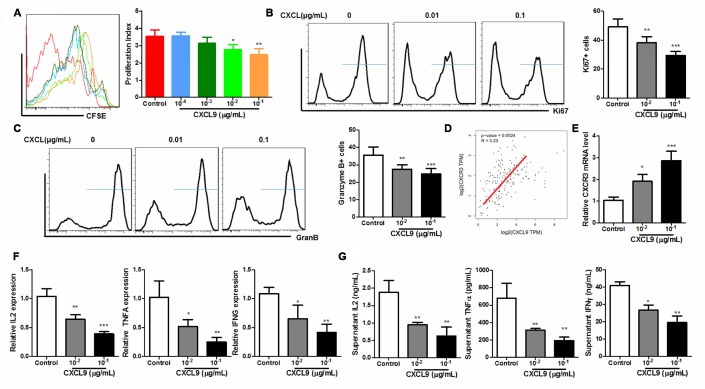
**CXCL9 directly inhibited *in vitro* activation of CD8+ T cells.** (**A**) showed that *in vitro* CXCL9 treatment dose-dependently repress division of activated CD8+ T cells; (**B**) showed that *in vitro* CXCL9 treatment significantly repressed expression of proliferation marker Ki67; (**C**) showed that *in vitro* CXCL9 treatment significantly repressed expression of activation marker Granzyme B; (**D**) showed a positive correlation between CXCL9 and CXCR3 expression in PAAD tissue; (**E**) showed that CXCL9 treatment significantly induced CXCR9 expression in CD8+ cytotoxic T cells; (**F**) showed that *in vitro* CXCL9 significantly suppressed the mRNA expression of anti-tumour cytokines IL2, TNFα and IFNγ in CD8+ cytotoxic T cells; (**G**) showed that *in vitro* CXCL9 treatment suppressed the secretion of anti-tumour cytokines IL2, TNFα and IFNγ in CD8+ cytotoxic T cells. *p<0.05, **p<0.01, ***p<0.001 when compared with control.

To further understand if supplementation of CXCL9 has a direct effect on CD8+ cytotoxic T cells, we isolated CD3+CD8+ T cells from splenocytes of naive C57BL/6J mice and activated the cells with anti-CD3 and anti-CD28 antibodies in the presence of different concentration of recombinant murine CXCL9. By measuring the CFSE proliferation profile of the cells, we found that CXCL9 could dose-dependently suppress the proliferation of CD8+ cytotoxic cells. At dose higher than 10ng/mL, CXCL9 significantly repressed cell proliferation (Figure 5A). Expression of Ki67 and Granzyme B was also potently reduced in CXCL9-treated CD8+ cytotoxic T cells (Figure 5B and 5C). As CXCL9 is the primary ligand of CXCR3 receptors, we further measured if CXCL9 treatment could activate CXCR3 expression and signalling. Expression of CXCL9 in the tumour tissues of PAAD patients correlated with its CXCR3 expression (Figure 5D) and treatment of CXCL9 could significantly induce the expression of CXCR3 in CD8+ cytotoxic cells (Figure 5E). These may suggest that CXCL9-induced T cell exhaustion may be associated with CXCR3 and its related signalling. Also, treatment of CXCL9 could dose-dependently reduced the mRNA expression and secretion of anti-tumour cytokines from CD8+ cytotoxic T cells, including TNFα, IL2, and IFNγ, further proving that CXCL9 treatment could lead to cytotoxic T cell exhaustion and dysfunction. These findings suggest that suppression of cytotoxic T cells *in vivo* should be attributed to the direction inhibitory action of CXCL9.

### STAT3 signalling was responsible for the CXCL9-induced cytotoxic T cell exhaustion and dysfunction

**Figure 6:**
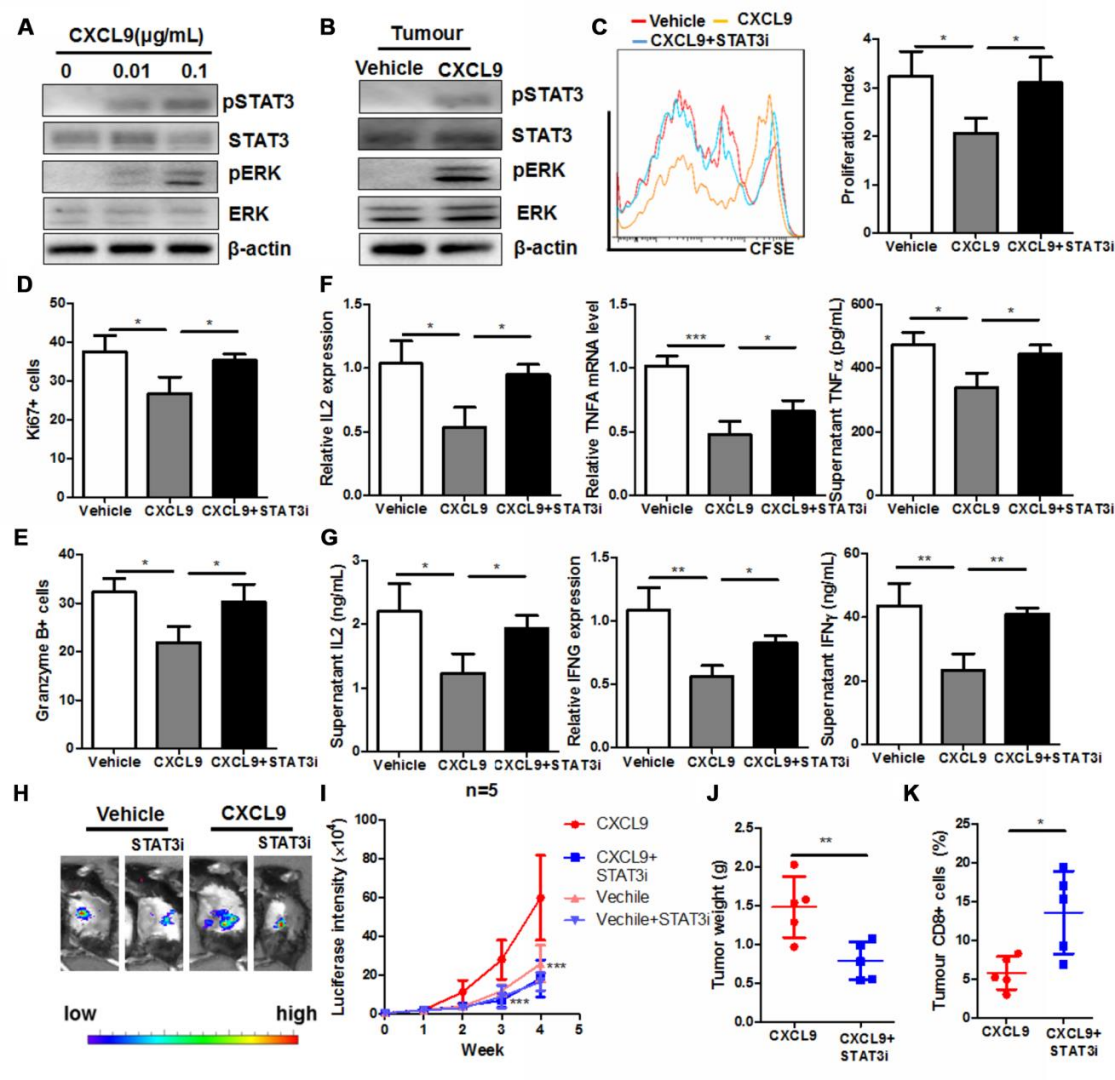
**STAT3 activation was responsible for the CXCL9-induced suppression of CD8+ T cell activation.** (**A**) showed that CXCL9 dose-dependently induces activation of STAT3 and Ras signalling in CD8+ cytotoxic T cells; (**B**) showed that CXCL9 treatment could induce STAT3 activity in PAAD tumour; (**C**) showed that inhibition of STAT3 by selective inhibitor recovered CXCL9-suppressed division of CD8+ cytotoxic T cells; (**D**) inhibition of STAT3 by selective inhibitor attenuated the suppression of Ki67 expression in CXCL9-treated CD8+ cytotoxic T cells; (**E**) inhibition of STAT3 by selective inhibitor attenuated the suppression of Granzyme B expression in CXCL9-treated CD8+ cytotoxic T cells; (**F**) inhibition of STAT3 by selective inhibitor recovered the mRNA expression of anti-tumour cytokines IL2, TNFα and IFNγ in CD8+ cytotoxic T cells; (**G**) showed that inhibition of STAT3 by selective inhibitor recovered the secretion of anti-tumour cytokines IL2, TNFα and IFNγ in CD8+ cytotoxic T cells. *p<0.05, **p<0.01, ***p<0.001 when compared with CXCL9 treatment only. (**H**) showed that presence of STAT3 selective inhibitor could signficantly reduce the tumour burden in mice; (**I**) showed that tumour growth induced by CXCL9 was abolished by STAT3 selective inhibitor; (**J**) showed that enlarged tumour weight induced by CXCL9 was abolished by STAT3 selective inhibitor; (**K**) showed that presence of STAT3 selective inhibitor could recover the CD8+ population in PAAD tumour.

It was recently observed that CXCL9 could activate Jak2/ STAT3 signalling in podocytes and correlates with retina inflammation in patients with diabetic retinopathy [28]. STAT3 activation in CD8+ cytotoxic T cells was responsible for the immune evasion in cancer patients [29]. Activation of STAT3 by CXCL9 was observed in CD8+ cytotoxic T cells in our study. Ras signalling was also activated in CD8+ T cells upon CXCL9 treatment (Figure 6A). CXCL9 treatment could significantly increase the STAT3 activity in the PAAD tumour of mice (Figure 6B). To understand if STAT3 activation was responsible for the CXCL9-induced cytotoxic T cell exhaustion and dysfunction, we treated CD8+ cytotoxic T cells with CXCL9 in the presence of selective STAT3 inhibitor cryptotanshinone (5μM). The presence of STAT3 inhibitor could significantly recover cell proliferation of CD8+ cytotoxic T cells in the presence of CXCL9 (Figure 6C). Suppression of Ki67 and Granzyme B expression in CXCL9-treated CD8+ cytotoxic T cells was attenuated by STAT3 inhibitor (Figure 6D and 6E). Furthermore, STAT3 inhibition potently recovered the expression and secretion of anti-tumour cytokines, suggesting the recovery of function of CD8+ cytotoxic T cells (Figure 6F and 6G). To understand the role of STAT3 in CXCL9-mediated tumour progression, we injected STAT3 inhibitor STATTIC (25 mg/kg, twice per week, intraperitoneally) to CXCL9-treated PAAD mice [30], and we found that compared with CXCL9-treatment only, STATTIC co-injection could significantly reduce the orthotopic tumour growth, and the infiltration of CD8+ cells into PAAD tumour was recovered (Figure 6H–6K). These data suggested that STAT3 signalling was responsible for the CXCL9-induced cytotoxic T cell exhaustion and dysfunction.

## DISCUSSION AND CONCLUSION

The role of CXCL9 in human cancers remains unclear and contradictory. It was found that CXCL9 was expressed in most types of human cancers, including breast cancer, hepatocellular carcinoma, melanoma, ovarian cancer, gastric carcinoma, cervical cancer, prostate cancer as well as glioma [31]. However, several studies reported a relatively low expression of CXCL9 in tumour tissues of colorectal cancer and non-small cell lung cancer [32, 33]. In cutaneous T-cell lymphoma, expression of CXCL9 was found at early stage but low at advanced stage [34]. Regardless its expression, both tumour-promoting and tumour-suppressive roles of CXCL9 were reported even in the same cancer type. In our study, we found that CXCL9 was overexpressed in PAAD tissues compared with healthy adjacent pancreas. Supplementation of CXCL9 in a murine PAAD model accelerated tumour progression, indicating that CXCL9 may exert a tumour-promoting role in PAAD.

Further study suggested that CXCL9 supplementation could defect the CD8+ cytotoxic T cells proliferation and function. Some previous studies have shown that CXCL9 exhibited both promoting and suppressive effects on CD8+ cytotoxic T cells. The strong positive correlation between CXCL9 and CD8 expression was found in prostate tumours [35]. Chow et al. found that CXCL9 facilitated the dendritic cell-CTLs interaction in the tumour microenvironment, which activated the CD8+ T cells response in cancer [36]. CXCL9, in this case, was secretion by the CD103+ dendritic cells and facilitated the recruitment of CD8+ cytotoxic T cells [37]. Inhibition of CXCL9 reduced CD8+ T cell infiltration into the tumour during the treatment of Listeria monocytogenes-based anticancer vaccines [38]. In contrast, it was found that CXCL9 was associated with reduced cytotoxic T cell infiltration during immunogenic chemotherapy [39]. In our study, we found that direct treatment of CXCL9 could induce exhaustion of CD8+ cytotoxic T cells, which led to reduced proliferation and infiltration of CTLs into PAAD tissue. Adoptive transfer of CD8+ cytotoxic T cells in CXCL9-treated mice suppressed tumour growth, further proving that CXCL9 delivers tumour-promoting effect through suppressing CTLs.

We found that activation of STAT3 may exhibit an immunosuppressive effect in PAAD. Previous studies have suggested that STAT3 activation is an essential modulator in the immunosuppressive microenvironment in PAAD. Hu et al. showed that STAT3 transcriptionally regulates the PD-L1 expression in PAAD tissues, which was considered as the significant executor of immune evasion in cancer [40]. Javeed et al. found that in CD14+ monocytes, STAT3 activation could induce arginase expression and reactive species oxygen to create an immunosuppressive microenvironment [41]. Activation of STAT3 could also re-polarize the tumour-associated macrophages towards immunosuppressive M2 phenotypes [42]. STAT3 activation in Activation of Jak2/STAT3 in dendritic cells raised the level of Th2 cytokines, which suppressed the CTLs in PAAD [43]. Lu et al. further suggested that the immunosuppressive action of STAT3 activation was due to the induction of chronic inflammation in the tumour microenvironment that can impair CTLs activation [44]. CD69+ Treg cells of PAAD tissue was responsible for the production of immunosuppressive cytokine IL10 [45]. Our study suggests that STAT3 activation in CD8+ effector cells could directly result in suppression of its proliferation, activation, and secretion of anti-tumour cytokines, indicating that STAT3 may regulate the tumour microenvironment of PAAD in multiple immune cells.

The data to construct Figure 3A was directly retrieved and analyzed by the platform TIMER (http://cistrome.org/TIMER/), which showed that CD8+ T cells were correlated with the survival of patients. Discrepantly, the data likely indicated that lower CD8+-cells predicted a better survival, which seems to be opposite to the data we showed in Figure 2. However, some previous studies have clearly shown that favour prognosis of high CD8+ T cells in pancreatic adenocarcinoma, in combination with the analysis of expression of other factors [46–48]. Interestingly, Fogar et al. showed that pancreatic cancer patients have significantly higher level of CD8+-cell infiltration into the pancreas than healthy control. This is reasonable because tumour cells will surely activate the T-cell defence system [49]. It was therefore predictable that a more massive tumour would attract more CD8+-cells than small tumour. As larger tumour usually is leading to lousy prognosis of PAAD patients, it would also be discussable whether the number of CD8+-cells could directly indicate a prognosis of PAAD patients [50]. Indeed, a recent study suggests that CD8 could not be a sole factor for predicting the prognosis of PAAD patients; other factors should be combined [51]. In our study, we find that CXCL9 treatment could reduce the expression of proliferation marker Ki67 and activation marker Granzyme B, suggesting that the role of CXCL9 in reducing CD8+ cells infiltration, as shown in Figure 2, was at least partially related to its regulation on CD8+-cell proliferation and activation. Hwang et al. recently showed that high CD8+Granzyme B+ cells in PAAD predicted favour patient prognosis [52], which could be consistently concluded from our observation. On the other hand, some other studies may support to explain the discrepancies of human samples retrieved from database with experimental data in mice model. Zhang et al., in their recent study, found that intratumoral CD8+-cells was even a poor prognosis of PAAD, while intraepithelial CD8+-cells predicted good prognosis [53]. This was consistently observed in study by Liu et al. in which peritumoral CD8+-cells in PAAD patients showed better prognosis [50], suggesting that sub-tissue localization of CD8+-cells may also have differential roles in predicting the survival of PAAD patients. As our studies collected the whole tissue of pancreas from mice to analyze the CD8+ populations, while human samples from TIMER database major analyzed intratumoral CD8+ cells, the conclusion may be different. Figure 3A majorly indicates the relevance of different immune populations with PAAD patient survival, while detail analysis by our study was performed for understanding its role in PAAD.

To understand if CXCL9 plays a role in the activation of RAS signaling in PAAD, we treated primary CD8+ T cells with CXCL9 and determined the Ras signaling. We found that CXCL9 could significantly activate Ras signaling in CD8+ T cells. This was consistent with a similar study in other cell types, in which Ras was shown as the down-stream signaling of CXCR3 activation, the receptor of CXCL9 [54]. The activation of Ras by CXCL9 in CD8+ T cells may play a pro-tumoral role, as it has been shown that Ras activation in CD8+ T cells inhibited its cytotoxic functions and therefore promoted tumour growth [55], while inhibition of Ras in tumour could potently enhance the reactivity of CD8+ T cells and blocked tumour progression [56, 57]. Reciprocally, Ras activation could also control the CXCR3 signalling through the modulation of gene expression and therefore may further enhance the pro-tumoral signalling [58]. It is so far not conclusive that CXCL9 could directly lead to Ras activation in PAAD, but from the observation by us and by aforementioned literature, CXCL9 may contribute to the unresponsiveness of anti-cancer treatment partially through the activation of Ras in CD8+ cytotoxic T cells, which leads to the loss of its anti-tumour function.

The limitation of our study is that we have not yet identified the origin of overexpressed CXCL9 in PAAD tissue. Several previous studies have found that multiple types of cells in tumour microenvironment could secrete CXCL9. Pascual-García et al. found that leukaemia inhibitory factor (LIF) could regulate CXCL9 expression in TAMs, which led to reduced infiltration of CD8+ T cells in brain tumours [59]. Yang et al. indicated that STAT3 signalling could regulate the expression of CXCL9/CXCR3 in T lymphocytes [39]. Interestingly, De Siva et al. reported that FOXP1 could initiate the CXCL9 expression in primary breast cancer cells [60]. Our analysis showed that CXCL9 expression had a negative correlation with the purity of tumour cells in the tumour microenvironment of PAAD, suggesting that CXCL9 in PAAD was not likely to be produced by tumour cells, but was possibly initiated by the stromal cells in the tumour microenvironment. In addition, CXCL9 was molecularly regulated by several transcriptional factors, including Jak/STAT1 and NF-κB [31]. The blockade of STAT1 activity or activation of NF-κB could initiate the expression of CXCL9 by a transcription-dependent mechanism [61]. Interestingly, it was found that IFNγ could block CXCL9 expression via activating STAT1 pathways [62, 63]. In our study we found that CXCL9 reduces the IFNγ expression in cytotoxic T lymphocytes. This may indicate that there was a positive feedback loop between the CXCL9 and IFNγ regulation in PAAD. The exact mechanism of CXCL9 regulation remains to be further investigated in the future.

In conclusion, in this study, we identified the expression pattern, clinical significance, and the role of CXC chemokines in PAAD. Three CXC chemokines, including CXCL5, CXCL9, and CXCL10, were significantly overexpressed in PAAD tissue compared with the adjacent healthy pancreas and were correlated with the poor survival of the patients. CXCL9/10 was associated with changed of immune cell pattern in the tumour microenvironment, and supplementation of CXCL9 in orthotopic murine PAAD model promoted tumour progression. In particular, CXCL9 reduced the CD8+ cytotoxic T lymphocytes in the tumour microenvironment of PAAD, which could be attributed to the reduced CD8+ T cell proliferation, activation, and secretion of anti-tumour cytokines. *In vitro* treatment of CXCL9 directly led to the suppression of the proliferation, activation, and secretion of anti-tumour cytokines of isolated CD8+ T cells. CXCL9 activated STAT3 signalling in CD8+ T cells, and inhibition of STAT3 recovered the proliferation, activation, and secretion of anti-tumour cytokines of CD8+ T cells. Our study indicates CXCL9 as a potential target of immunotherapy in PAAD treatment by regulating the CD8+ T lymphocytes in the tumour microenvironment.

## MATERIALS AND METHODS

### Chemicals and reagents

Recombinant murine CXCL9 was purchased from Peprotech (USA). Selective STAT3 inhibitor cryptotanshinone was purchased from Selleckchem (USA). Anti-CD3ε and anti-CD28 antibodies were purchased from Biolegend (USA). Antibodies against phosphor-STAT3, STAT3, and β-actin were purchased from Cell Signalling Technology (USA). STATTIC was purchased from Sigma-Aldrich (USA).

### Orthotopic PAAD model

The protocol of animal study was approved by the Ethic Committee of the Department of Laboratory Animal Science, Fudan University (No. 20160673A040; Shanghai, China). The orthotopic PAAD murine model was established with modification from our previous study [22]. In brief, 1×10^6^ murine PAAD Panc-2 cells expressing luciferase reporter were mixed with same volume of Matrigel Matrix (BD Bioscience, USA) and were injected into the pancreas of C57BL/J mice. One week after tumour cells injection, the orthotopic PAAD tumour was examined under Live animal imager by intraperitoneally injecting 15mg/kg luciferin. Mice with comparable levels of luciferin signals were included in the studies. For the flow cytometry analysis, PAAD tissue was minced into small pieces and digested in collagenase IV (0.8mg/mL) for 45min at 37°C with gentle shaking. Samples were then filtered with 70μM cell strainer and spin down. The cell pellet was then resuspended in ACK buffer for 5min and then proceed for staining of cell surface marker for analysis.

### Flow cytometry-activated sorting

Splenocytes were isolated, and red blood cells were lysed with ACK buffer (BD Bioscience, USA). Cells were then stained with anti-CD3 and anti-CD8 antibodies (Biolegend, USA). CD3+CD8+ cells were then sorted on AriaI Cell Sorter (BD, Bioscience, USA).

### *In vitro* T cell activation assay

Flat bottom 96-well cell culture plate was coated with 5μg/mL anti-CD3 antibody at 4°C overnight. 5×10^5^ sorted CD3+CD8+ cells were resuspended in 200μL RPMI1640 medium supplemented with 10% FBS and added to each well. 2μg/mL anti-CD28 antibody was added. T cells were activated for 72h before detection.

### Adoptive T cell transfer

CD8+ cytotoxic T cells were isolated from splenocytes of naive mice and activated *in vitro*. 1×10^7^ activated cells were resuspended in HBSS and were injected intravenously into the orthotopic PAAD mice. Another adoptive transfer was performed two weeks after first transfer to maintain the *in vivo* level of CD8+ cytotoxic T cells.

### Carboxyfluorescein succinimidyl ester (CFSE) and intracellular staining

For CFSE labelling, CD3+CD8+ cells were resuspended in 1mL PBS containing 2μM CSFE and stayed in the dark for 25min. Labelled cells were then washed with PBS and proceeded for *in vitro* activation assay. For intracellular staining, activated T cells were fixed and permeabilized with Fixation and Permeabilization Solution (BD Bioscience, USA) for 1h followed by the incubation with anti-Ki67 and anti-Granzyme B antibodies (Biolegend, USA) for 30 min. Cells were then washed before detection.

### Quantitative real-time PCR

Total RNA was extracted with Trizol reagent (Thermo Scientifics, USA). Template cDNA was prepared with a First-strand synthesis kit (Takara, Japan). Gene expression was then measured by SYBR Green assay (Takara, Japan) on LC480 platform (Roche, USA) using the primers as shown in Table 1.

**Table 1:** Primers for quantitative real-time PCR.

**Gene**	**Forward (5′→3′)**	**Reverse (5′→3′)**
*IL2*	CCTGAGCAGGATGGAGAATTACA	TCCAGAACATGCCGCAGAG
*TNFA*	CTGTAGCCCACGTCGTAGC	TTGAGATCCATGCCGTTG
*IFNG*	TCAAGTGGCATAGATGTGGAAGAA	TGGCTCTGCAGGATTTTCATG
*β-actin*	AGAGGGAAATCGTGCGTGAC	CAATAGTGATGACCTGGCCGT

### Immunoblotting

Total protein was extracted with RIPA buffer (Sigma-Aldrich, USA) and separated on SDS-PAGE (Biorad, USA). Protein was then transferred onto PVDF membrane (Roche, USA) and blocked with 5% BSA in TBST at room temperature for 2h. Primary antibody incubation was performed at 4°C overnight, followed by secondary antibody incubation for 2h at room temperature. Expression of related protein was read with ECL select substrate (GE Healthcare, Germany) on ChemiDoc system (Biorad, USA).

### ELISA assay

Expression of IL2, TNFα, and IFNγ in mouse serum or cell culture supernatant was measured by ELISA assay kit (R&D Systems, USA) according to the manufacturer’s instruction.

### Statistical analysis

The comparison was made with student T-test between two groups, while One-way ANOVA was applied when three or more groups were compared. p < 0.05 was considered statistically significant.
